# Remote Metastasis of Oral Squamous Cell Carcinoma to Cavernous Sinus: A Report of a Rare Case

**DOI:** 10.7759/cureus.27373

**Published:** 2022-07-27

**Authors:** Anendd Jadhav, Aishwarya Gupta, Nitin Bhola, Hardik Karia

**Affiliations:** 1 Oral and Maxillofacial Surgery, Sharad Pawar Dental College and Hospital, Datta Meghe Institute of Medical Sciences, Wardha, IND

**Keywords:** squamous cell carcinoma., metastasis, human papilloma virus, facial paralysis, cavernous sinus

## Abstract

The most frequently occurring malignant tumor of epithelial origin of the head and neck region is squamous cell carcinoma (SCC). It is characterized by loco regional dissemination whilst remote metastasis (RM) is rare. The lung, bone, and liver are the frequent sites for RM whilst involvement of the brain or cavernous sinus has an exceptionally rare occurrence. Owing to its rarity, lack of awareness amongst head neck surgeons, and absence of any evidence-based protocol, the optimal management strategies in this population are controversial and, hence, associated with dismal outcomes. The present case report exhibits a rare presentation of cavernous sinus metastasis in human papillomavirus (HPV)-related primary SCC arising from the lower gingivobuccal complex.

## Introduction

Loco-regionally advanced oral squamous cell carcinoma (OSCC) is characterized by remote metastasis (RM). The frequent sites of RM are lungs, bone, and liver although other sites such as the brain and spine are also reported [1]. The estimated prevalence of brain metastasis alone and with metastases at remote sites are indexed at 0.4% and 2-8% respectively [2]. There is growing evidence of human papillomavirus (HPV)-associated OSCC having a propensity for RM at atypical sites [3]. Although its association is considered a prognostic marker, this does not preclude brain metastasis. The present case is exhibited with an intent to envisage the head neck surgeon regarding the atypical and rare presentation of cavernous sinus metastasis (CSM) from HPV-related gingivobuccal sulcus OSCC.

## Case presentation

**Figure 1:**
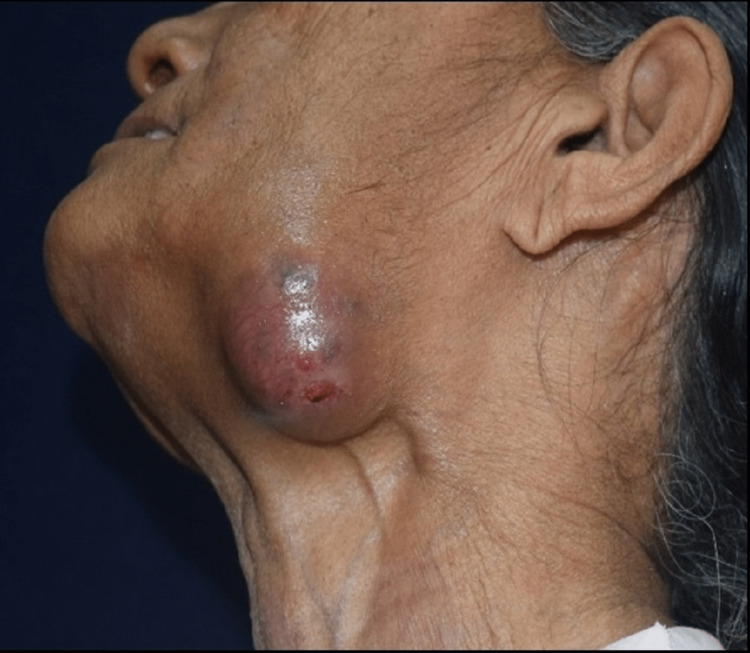
Secondaries in homolateral submandibular gland

A systemically healthy, 53-year-old female from the countryside presented with a presence of painful, progressive ulceration at lower left posterior region of the jaw for five months. Anamnesis reported a recent onset of tearing and acute loss of visual acuity with spontaneous facial weakness on left side. Reduced inter-incisal opening did not permit detailed intraoral examination. Extraorally, there was single, diffuse swelling measuring 5 x 3 cm in the greatest dimension over the left submandibular region, with upper and mid-jugulo-digastric lymphadenopathy (Figure 1).

**Figure 2:**
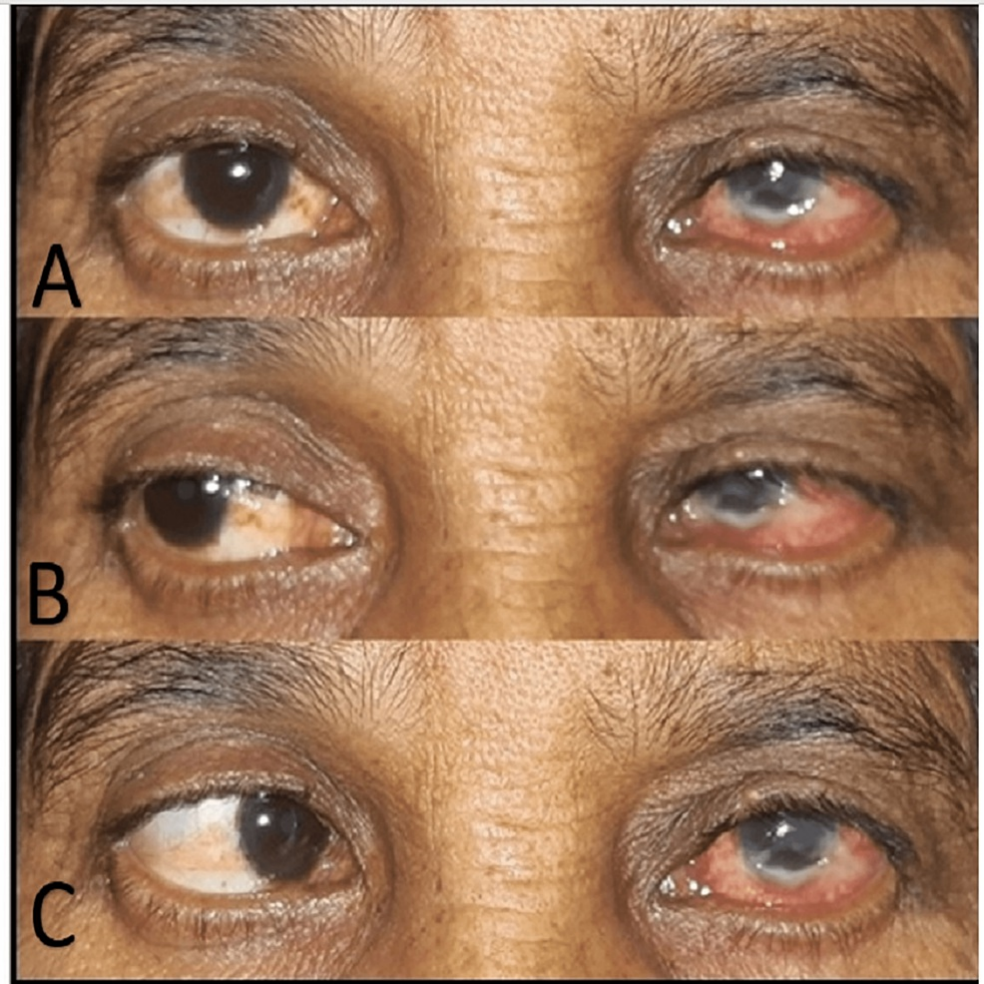
(A) Chemosis of left eye, (B) Unrestricted movement of left eye on medial side, (C) Left Esotropia with major limitation of abduction showing left VI (abducens) nerve paralysis

Clinical evaluation revealed generalised facial nerve weakness of left side with chemosis, corneal opacification, acute loss of visual acuity, and partial external ophthalmoplegia and esotropia with the left eye (Figure 2).

**Figure 3:**
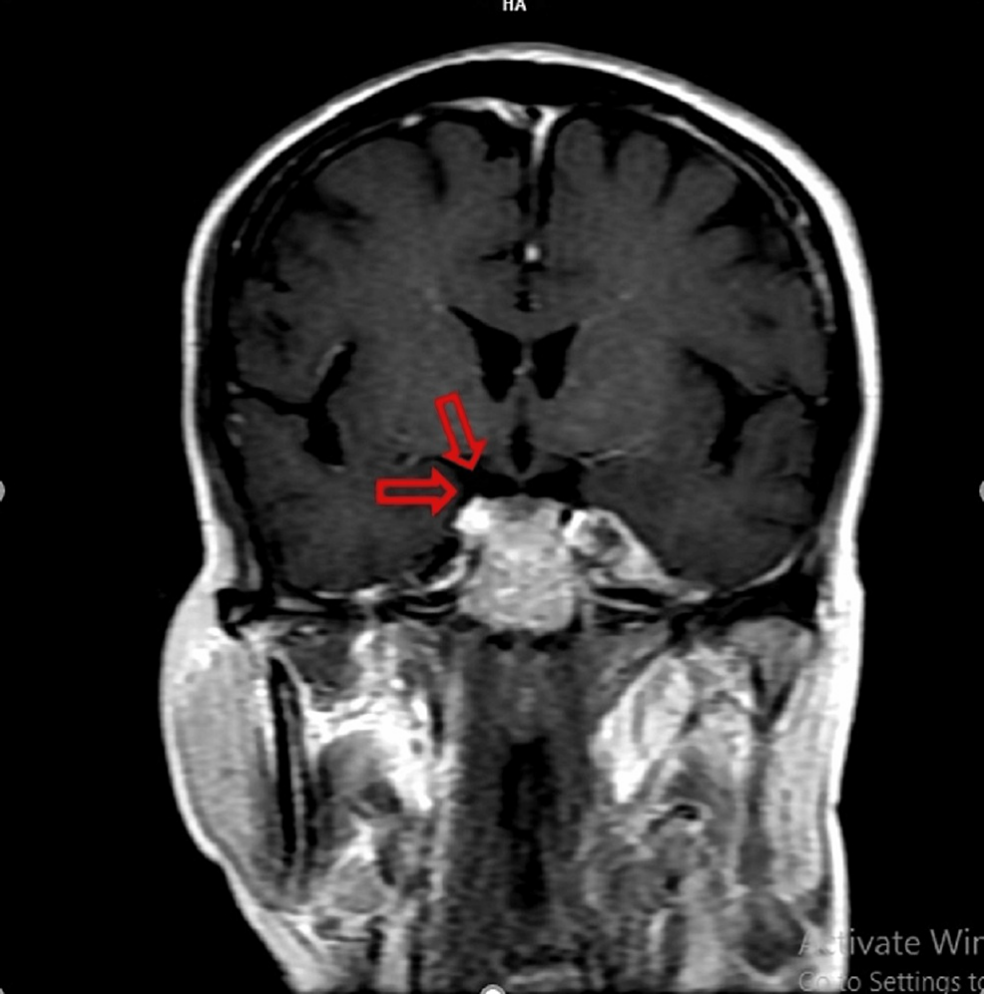
MRI brain (coronal view) showing intense post-contrast enhancement (arrow) in left cavernous sinus s/o metastasis

Medical records confirmed HPV-related OSCC using P16 immunohistochemistry testing. The findings led us to suspect intracranial involvement for which MRI brain was demanded which confirmed presence of two lesions in cavernous sinus, which shows mild to moderate enhancement on post contrast study. The lesion on the left cavernous sinus (CS) encased the left internal carotid artery extending to involve the sella. On the contralateral side, the lesion involves the right CS and clivus at the level of Dorello's canal (Figure 3).

A whole-body MRI metastatic work-up was unremarkable. Palliative management with chemo-radiotherapy was advised. The patient succumbed to the disease after two months of therapy.

## Discussion

There is an emerging trend of HPV-related OSCC without active HPV infection. HPV-related OSCC exhibits a propensity for RM to unusual sites, especially to the brain, dura mater, and bone; however, a contradictory view has also been reported [4-7]. The involvement of CS has a typical presentation of those chiefly related to cranial nerves III, IV, ophthalmic branch of V, VI involvement [7]. The carotid artery and III-VI CN are seen traversing it. CS receives blood from orbital veins and drains into petrosal sinuses. Therefore, diplopia, ophthalmoplegia, orbital congestion, reduced corneal reflex, dysesthesias, retro-orbital pain, hypesthesia, headache, and varying degrees of facial pain may be present [6]. The involvement of CN VII and ischaemic vasa nervosum causes pain and alteration of motor and sensorineural function whilst other symptoms such as ophthalmoplegia are attributed to the involvement of CN III-VI [8]. Our patient presented with signs of involvement of CN VI and VII like lateral rectus palsy, chemosis, left esotropia, ipsilateral facial paralysis, and headache.

CN VI involvement can also be seen in tumors of the pituitary gland laterally extending to involve CS; however, the lack of any other neuro-endocrinologic markers obviates its presence. The CSM may be attributed to the neurotropism of OSCC, direct extension of the primary tumor, or to the hematogenous spread. In the case exhibited, the direct extension of the primary tumor and perineural spread was precluded by the absence of the disease extension by MRI.

MRI helps in precise delineation of the presence of solid tumors and blood in the CS and, hence, was the preferred modality in the present case. Histological confirmation is desirable; however, diagnostic surgical exploration is challenging and associated with high morbidity [9]. Therefore, in the present case, the diagnosis was based on clinical and MRI findings. Conditions that resemble CSM include intracavernous carotid fistula, aneurysm, pituitary carcinoma, meningioma, and cavernous sinus thrombosis.

The treatment of CSM comprises surgery, radiotherapy, and chemotherapy chiefly with a palliative intent as the expected two-year mortality rate is 75-85% [10]. With the intent of eradicating the metastatic disease, curative intent treatment by surgery or radiation can still be a viable option. The role of stereotactic radiosurgery in treating brain metastasis is appealing lesions located in eloquent areas measuring < 2.5 cms lacking any considerable mass effect [1]. Gamma knife radiosurgery is an emerging concept in the surgical management of the symptomatic disease and has demonstrated reduction of the radiotherapy dose, enhanced tissue sparing effect, and thereby minimizing the toxicity [5].

The role of chemotherapeutic agents is speculative as most of them demonstrate poor blood-brain barrier penetration. High-dose cisplatin, 5 fluorouracil, and cetuximab followed by long-term cetuximab therapy have been recognized as first-line therapy with improved survival. However, their use soon fell out of favor due to the increased frequency of adverse effects such as myelosuppression, renal failure, and lack of literature support [10].

Palliative external beam radiotherapy remains the mainstay of treatment. However, site, size, dose, risk of adjoining areas, and extent of CN involvement are the major limiting factors [2]. Radiotherapy can be administered as external beam radiotherapy (20-30 Gray) or as radiosurgery (15 - 20 Gray) if the primary is in control. External beam radiotherapy has been known to provide good palliative radiation in symptomatic patients. With ablative radiotherapy, higher doses can be delivered compared to prolonged conventional radiotherapy. It demonstrates minimal adverse effects and toxicity and has a pronounced tissue salvage effect. Radiotherapy has demonstrated median survival of three to six months in comparison to one month (mean survival) for untreated patients [2,10].

T-stage primary located in the pharynx, second primary tumors, local disease control (absence of active disease), and burden of nodal disease may be independent risk factors for RM. Despite all treatment options, the prognosis remains poor. The patient in the exhibited report succumbed to the disease within two months of external beam radiotherapy. 

## Conclusions

The present case highlights the atypical presentation of RM of primary OSCC to CS in confirmed HPV status patients. There is a severe dearth of literature regarding CSM from a gingivobuccal malignancy. CSM is a rarity with the presenting symptoms chiefly related to the nerves involved. Pallative radiotherapy is the mainstay of management, however, with poor outcomes.
